# What Really Works? Testing Augmented and Virtual Reality Messaging in Terrestrial Invasive Species Management Communications to Impact Visitor Preferences and Deter Visitor Displacement

**DOI:** 10.1007/s00267-023-01787-z

**Published:** 2023-01-16

**Authors:** Ingrid Schneider, Brett Rannow, Angela Gupta, Matt Russell, Marcella Windmuller-Campione

**Affiliations:** 1 Department of Forest Resources, University of Minnesota - Twin Cities, St Paul, MN USA; 2 University of Minnesota - Twin Cities, Extension, Rochester, MN USA

**Keywords:** Emerald ash borer, Advanced communication technologies, Stakeholder

## Abstract

Natural resource management is rapidly shifting to incorporate a deeper understanding of ecological processes and functioning, including attention to invasive species. The shift to understand public perceptions of resource management and invasives is much slower. Information influences both landscape preference and behaviors. Theory suggests that increasingly engaging information should have concurrently greater impacts. This research tested the effect of increasingly engaging information on visitor preferences and intentions to return to landscapes treated in response to emerald ash borer (EAB; *Agrilus planipennis*). Park visitors in a midwestern-U.S. state randomly received one of four messages about forest management in response to EAB (control, photo, augmented reality (AR) and virtual reality (VR)). Messaging impacted preferences for three of the four management approaches, but significant changes in displacement intentions emerged in only one of the four. Specifically, VR and AR increased preferences for complete harvest compared to photos/text, but not differently from those who received no information. VR significantly lowered preferences for select harvest with natural regeneration. The photo/text treatment increased preference for select harvest with planted trees over no information. Any information reduced displacement in response to a photo depicting “select harvest, planted trees.” Subsequently judicious use of advanced communications like VR can optimize increasing scarce resources and maintain or optimize ecological services. Future research directions across geographic and content areas are recommended.

## Introduction

Natural resource management is rapidly shifting to incorporate a deeper understanding of ecological functioning, including invasive species and their management. The integration of stakeholder perceptions to invasive species management and policy is “vital” (Binimelis et al. 2008) as is stakeholder buy-in (Omondiagbe et al. 2020; Perry and Perry 2008; Shackleton et al. 2019). However, research on stakeholder perceptions of invasive species and their management remains an emerging area. There has been more research activity since the mid-2010s (García-Llorente et al. 2011; Kapitza et al. 2019; Shackleton, Richardson et al. 2019) and a 2019 literature review revealed that most public engagement around invasive species management is limited and one-way (Shackleton et al. 2019).

Terrestrial invasive species can negatively impact environmental esthetics. Insect invasions can kill trees and result in dead wood, open forest canopies, and dense understories. Among such invasive species is the emerald ash borer (EAB, *Agrilus planipennis)*. EAB is one of the most damaging and costly forest-borer insects to establish in the United States since its 2002 introduction (Aukema et al. 2011; Marzano et al. 2020; University of Minnesota Extension 2019) and, internationally, EAB’s presence in Russia increases the risk of incidental transportation into other areas in Asia and Europe (Marzano et al. 2020; Orlova-Bienkowskaja and Bieńkowski 2022).

Tree health issues are an ongoing and increasingly concerning public issue, especially for those who enjoy and recreate in forests (Urquhart et al. 2018). Forest landscape changes caused by invasive species impact esthetics, outdoor recreation, and nature-based tourism (Filyushkina et al. 2017; Lee 2001; Ribe 1989; Robertson and Regula 1994; Schneider et al. 2019). Outdoor recreation is a critical ecosystem service (Daniel et al. 2012; Filyushkina et al. 2017; Slee 2005) that contributes nearly $375 billion (1.8%) to the U.S. Gross Domestic Product (GDP; Bureau of Economic Analysis 2021). Visitors seek esthetically pleasing landscapes (Driver and Brown 1986; Hendee et al. 1971; Palmer and English 2019; Sotomayor et al. 2014) and environmental managers consider esthetic values, especially near high-visitor volume areas (Kearny 2001; Kovacs et al. 2006; Ribe 2002; Stankey and Clark 1992), through visual management frameworks (Bureau of Land Management 1984; National Park Service 2016; US Forest Service 1996; Washington State Department of Natural Resources et al. 2017).

Not only do invasive species impact environmental esthetics, but so do management actions to address them. In the case of EAB, forest management can leave behind canopy gaps, produce harvest residuals, and expose bare soil or tree trunks. These impacts are both negatively perceived (Arnberger et al. 2017; Edwards et al. 2012; Gundersen and Frivold 2008; Ribe 1990; Verlič et al. 2015) and may lead to visitor displacement. In turn, communities may lose tourism revenue and visitors may have sub-optimal experiences (Arnberger et al. 2018; Flint et al. 2009; Robertson and Regula 1994; Schneider et al. 2019).

Public information may improve understanding of and support for management actions (Eriksson et al. 2019; Garcìa-Llorente et al. 2011; Kearney 2001; Pierskalla et al. 2007; Ryan 2012; Sharp et al. 2012; Sumner and Lockwood 2020). Research reveals that increasingly engaging information has a greater impact on attitudes, behavioral intentions, and behaviors than less-engaging information (Ahn et al. 2014; Alyahya and McLean 2021; Cai 2013; Marto et al. 2019). While available and increasingly of interest (National Park Service 2020; Winter et al. 2020), the impact of engaging information technologies in environmental and forest management remains understudied. In response to calls for expanded research on the role of information in invasive species perceptions (Rodríguez-Rey et al. 2022; Ryan, 2012; Schlueter and Schneider 2016; Schneider et al. 2019), this project addressed if and how increasingly engaging information impacts visitor landscape preferences and return-visit intentions in response to select management strategies.

### Forest Landscape Preferences

Multiple models exist to understand and predict landscape preferences. Landscape models suggest preferences vary based on several factors. Specifically, among samples in the U.S. southwest, increased beauty emerged with greater tree density, average tree diameter, and crown-canopy cover (Daniel and Boster 1976). Among samples in the United States and beyond, perceived beauty increased with open forest structures, big trees, the presence of ground vegetation, and species variety (Hegetschweiler et al. 2017; Ribe 1990). Beyond esthetics, psycho-evolutionary models concurred visitors prefer structurally open, biologically diverse forests that have unrestrictive ground vegetation, moderate to high complexity, moderate to high depth, homogenous ground surface texture, no appraised threats, and water (Daniel and Boster 1976; Kaplan and Kaplan 1989; Kaplan et al. 1989; Ribe 1990; Ulrich 1983).

Undesirable forest landscape features include evidence of death like standing or downed dead wood, slash (Arnberger et al. 2017; Daniel and Boster 1976; Daniel and Schroeder 1979; Ribe 1990; Ryan 2005; Schneider et al. 2019), mono-species stands, immature forest stands, too much or too little canopy coverage, high densities of small trees, highly dense understory vegetation, and exposed bare soil (Daniel and Boster 1976; Edwards et al. 2012; Filyushkina et al. 2017; Gundersen and Frivold 2008; Ribe 1989, 1990).

Invasive infestations produce a “wide variety of visual effects depending on the forest type, the specific pest, and many other factors including the temporal stage and biophysical conditions” (Sheppard and Picard 2006, p. 325). Particular to EAB, once the insect establishes in a tree, its crown’s foliage thins, cracks appear on the tree’s trunk, and numerous woodpecker holes evolve. Mortality occurs quickly; an individual tree can be dead within 2 years and a whole ash forest can be impacted in a decade (Hahn 2021; Marshall 2020). In terms of management practices, large cuts (>15 acres-Haakenstad 1972), logging residue and left-behind trunks are typically disliked by the public and can negatively impact recreation experiences (Arnberger et al. 2017; Edwards et al. 2012; Gundersen and Frivold 2008; Hollenhorst et al. 1991; Ribe 1989; Ryan 2005; Schroeder et al. 2010).

Despite these impacts, limited existing research reveals visitors generally accept and support invasive species management (Schlueter and Schneider 2016) or management which addresses episodic endemic species infestations (McFarlane and Watson 2008). Park visitors found five out of eight management treatments in response to EAB acceptable, regardless of application in natural or use areas: wood regulations, sanitation cutting, progressive thinning, biological control, and creating sinks. Additionally, six out of eight were significantly more acceptable when applied in “use” areas compared to “natural” areas (Schlueter and Schneider 2016).

While visitors may accept and support forest management in response to EAB, their preferences for the resultant landscapes vary. In the limited research on visitor perceptions in this area, Arnberger and colleagues (2017, 2019) found the importance of the ash-forest and its management was important, particularly the foreground, but this importance varied across populations and sites: urban-forest visitors in the United States perceived EAB and management attributes more important to visual preferences than those in Austrian urban-forests (2019) and also preferred dense trailside vegetation and landscapes with at least some trees. European visitors indicated social factors played a larger role than landscape conditions (Arnberger et al. 2019). Earlier work on bark beetles (MPB; *Dendroctonus ponderosae* and *Ips typographus*) revealed visitors generally preferred any management over inaction (Arnberger et al. 2018; McFarlane and Watson 2008). In contrast, Müller and Job (2009) found tourists weakly unsupportive of management in response to bark beetles.

### Visitor Displacement

If conditions are negatively appraised, visitors may experience stress and respond in a variety of ways (Johnson and Dawson 2004; Kay and Jackson 1991; Kuentzel and Heberlein 1992; Schneider and Hammitt 1995; Schneider and Wilhelm Stanis 2007; Schneider and Wynveen 2015). One such response is displacement (Anderson and Brown 1984; Becker 1981). Visitors temporally displace when they change the time of a visit whereas spatial displacement finds visitors moving within an area (intrasite) or leaving an area altogether (intersite) (Hall and Shelby 2000; Kuentzal and Heberlein 1992). Most displacement research focuses on social (i.e., Hall and Shelby 2000; Rice et al. 2019) or managerial conditions (i.e., Schneider and Budruk 1999; Peden and Schuster 2009) that incite displacement with less research emphasis on environmental conditions. Environmental conditions are of primary interest in this study.

Displacement research in response to terrestrial landscape conditions is extremely limited. In the 1990s, Hollenhorst et al. (1991) found that with 30% or more canopy infestation, visitors were displaced from forests invaded by spongy moth [formerly known as gypsy moth; *Lymantria dispar*]. Schneider et al. (2019) found landscape changes caused by bark beetles, such as standing dead trees, interfered with up to 80% of respondents’ experiences and 70% of respondents intended to displace from an area heavily impacted by the beetle.

Beyond site conditions, certain visitor characteristics impact displacement, including experience or visitation. Repeat visitation relates to site condition sensitivity (Eder and Arnberger 2012; White et al. 2008; Urquhart et al. 2018). Therefore, first-time visitors are less sensitive to impacts and should be less likely to displace in response to undesirable environmental conditions (White et al. 2008). Indeed, studies reveal local, experienced visitors are more likely to engage in temporal displacement over spatial displacement in response to crowded conditions (Arnberger and Brandenburg 2007; Hansen et al. 2022; Manning and Valliere 2001). However, Peden and Schuster (2009) found that, in wilderness environments, limited relationships existed between visitation and intersite displacement.

### Influencing Visitor Preferences and Displacement Through Information

Despite the increasing frequency and significant impacts resulting from forest pests, the public is generally uninformed about them (Urquhart et al. 2018). With more information, visitors typically exhibit greater preference for the landscape they were informed about (Hanley et al. 2009; van der Wal et al. 2014), increased support for management (Jensen 2000; Ryan 2012; Tyrväinen et al. 2003) and reduced displacement.

Information to increase knowledge and familiarity with topics like invasive species management can influence both management perceptions (Novoa et al. 2017) and acceptance (Eriksson et al. 2019). Informational materials, such as brochures, are an important component of campaigns and are especially helpful in educating stakeholders with primary school or lower education levels (Zeng et al. 2021). Direct experiences can also raise awareness of environmental issues and influence a visitor’s understanding of and behavioral response to them (Brownlee et al. 2013). Experiencing negative impacts of invasive species increased public support for management, regardless of prior attitudes towards the invasive species (Fraser 2006; Loker et al. 1999). Beyond awareness, both general and specific knowledge about invasive species positively influenced support for invasive species management (García-Llorente et al. 2011; Pissolito et al. 2020; Sharp et al. 2011). In contrast, Müller and Job (2009) found that knowledge about native spruce bark beetles (*Ips typographus*) was strongly associated with decreased support to manage forests in response to them. Specific to forest management, knowledge of management projects, terms, and processes positively influenced support for disturbance-based management treatments (O’Brien et al. 2021; Shindler and Mallon 2006). Thus, the application of knowledge and familiarity related to EAB infestations seem warranted.

### Effective Informational Presentations

Natural area visitors most frequently receive information through static content like pamphlets and signs (Guo et al. 2015). While such static information can improve participant knowledge levels (Guo et al. 2017; Sharp et al. 2012; Young and Witter 1994), their persuasive influence varies (Guo et al. 2017; Price et al. 2018). The potential to influence attitudes and behaviors requires effective message design and presentation (Young and Witter 1994) that incorporates personal relevance and results in greater message elaboration (Petty and Cacioppo 1986). The Elaboration Likelihood Model explains this process (ELM; Petty and Cacioppo 1986).

The ELM presents two persuasion routes: 1) central and 2) peripheral. Central-route persuasion occurs when the recipient’s motivation and ability to cognitively elaborate on a message is high. Recipients utilize a diverse range of prior knowledge and issue-related thoughts to evaluate an argument through the central route. In contrast, peripheral-route persuasion occurs when the recipient’s motivation and ability to elaborate are low and they rely on peripheral cues to form judgments. Attitude changes via the central route tend to “show greater temporal persistence, greater prediction of behavior, and greater resistance to counter persuasion than attitude changes that result mostly from peripheral cues” (Petty and Cacioppo 1986, p. 5).

Of particular interest within the model are interactivity and engagement. Interactivity encourages message engagement and elicits greater central-route elaboration. Steuer (1992, p.84) defines interactivity as the “extent to which users can participate in modifying the form and content of a mediated environment in real-time. For example, website browsing among different persuasive messages can influence behaviors (Sundar et al. 2003; Xu and Sundar 2014). Oh and Sundar (2015) found message interactivity enhanced elaboration, led to greater cognitive absorption, and contributed to favorable attitudes towards both the medium and topic. Message engagement includes involvement with message content (Shin 2019) or attention to narrative and content (Bitgood 2009). In a persuasive context, Oh and Sundar (2015, p.215) define engagement as “a psychological state where users are either cognitively or emotionally involved in a task at hand.” Clearly, increasingly interactive messages require more cognitive effort to process.

Technology enhances engagement and persuasiveness via virtual immersion. For example, Fonseca and Kraus (2016) found the level of immersion within a virtual environment positively influenced a message’s emotional impact and recipients’ environmental attitudes. Two features define immersion: social presence and telepresence. Social presence is the “extent to which other beings, living or synthetic, exist in a virtual environment” (Schuemie et al. 2001, p.184) and can include hearing spoken audio or seeing a person within the virtual environment. One such example includes using conversational human voices in a tour to elevate visitors’ overall experience (Kang and Gretzel 2012). Telepresence “determines the degree of users’ immersion in a virtual environment, which can shape… information-gathering efficiency” (Nowak and Biocca 2003; Steuer 1992; Ying et al. 2022, p.1739). Determined by sensorially-rich environments and interactivity (Steuer 1992), telepresence tends to play a larger role in revisit intentions than social presence (Ying et al. 2022).

Technological advances have made creating interactive and engaging messaging easier, more affordable, and more accessible than before. Augmented reality (AR) enriches reality and “allows the user to see the real world, with virtual objects superimposed upon or composited with the real world” (Azuma 1997, p. 2; Klopfer and Squire 2008). AR is relatively accessible, as users can download AR applications on their personal devices and use them onsite (Chung et al. 2018; Harley et al. 2020), which they prefer (Pascoal et al. 2018). As a result, learning experiences are enhanced and reflected via desired attitude and behavioral changes (Cai 2013; Howard 2017). AR use elevates overall tourist experiences (Harley et al. 2020; Marto et al. 2019), producing more positive attitudes about a destination and stronger revisit intentions (Chung et al. 2018; Jung et al. 2015). Moreover, AR can help bridge the knowledge gap between experts and non-experts in landscape decision-making (Ghadirian and Bishop 2008). Beyond AR, virtual reality (VR) is “a simulated environment in which a perceiver experiences… presence by means of a communication medium” (Steuer 1992, p.76). Virtual environments are most typically viewed via headsets paired with a phone or computer that are worn over their eyes. VR immerses the user in the virtual setting, completely overtaking their field of vision and reacting to their movement; for example, when a user turns their head in real life, they also “turn” their head in the virtual environment. VR users can freely view the environment and, depending on the application, interact with it. VR has been recognized as a powerful tool in landscape planning (Orland et al. 2001) and has the potential to promote environmental literacy, garnering support for forest management (Chandler et al. 2021; Fauville et al. 2020; Qi et al. 2004).

A variety of studies have explored virtual landscape visualizations’ role in influencing responses to environmental issues (Burch et al. 2009; Sheppard et al. 2008; van Lammeren et al. 2010). Specific to recreation and tourism, high levels of VR immersion elicited significantly stronger revisit intentions than low-immersion VR (Ying et al. 2022). An early exploration of virtual forest development (Qi et al. 2004, p.4862) argued that a combination of VR, computer-supported cooperative work, and remote sensing technology “is a feasible and innovative way to support forest management.” Still, more than a decade later, a paucity of work examines the impact of interactive immersive messaging on landscape preferences and displacement in response to forest management. We explore this immersion and hypothesize that experiencing more interactive and immersive messages is positively related to preferences and negatively related to displacement intentions, regardless of landscapes.

## Methodology

### Study Sites

During the summer of 2021, onsite visitor questionnaires were completed at three Minnesota State Parks. Located in the Minneapolis-St. Paul (MSP) metropolitan area and along the confluence of the Minnesota and Mississippi Rivers, Fort Snelling State Park lies on the lands of the Dakota. The 1500-hectare state park hosts more than 700,000 annual visitors (Minnesota Department of Natural Resources 2022), is intended for day use, and accessed via personal vehicles, regional trails, and public transportation. Recreation opportunities available include hiking, non-motorized boating, fishing, and biking. Ash trees compose between 5 and 30% of the park’s forests (primarily green ash, *Fraxinus pennsylvanica*; Arnberger et al. 2017). EAB was discovered in the park in 2012 personal communication. E. Quinn, 2022.

Wild River State Park is located 58 miles (93 km) from MSP on the lands of the Ojibwe and Dakota. The 2750-hectare park hosts more than 230,000 visitors per year and is adjacent to 18 miles of the Wisconsin border along the Saint Croix National Scenic Riverway. Both non-motorized activities and overnight camping are primary recreation opportunities (Minnesota Department of Natural Resources n.d.). The park is accessible via personal vehicles, two river landings, and a bike trail. Pine, hardwood, and oak savannah forest ecosystems exist at the park and a mix of black (*Fraxinus nigra*) and green ash covers nearly 44% of it (Schlueter and Schneider 2016). As of 2022, EAB had not been detected within the park.

Lake Bemidji State Park is also on the lands of Dakota and Ojibwe, directly adjacent to Lake Bemidji, seven miles (11 km) from the town of Bemidji and nearly 200 miles from MSP. The park hosts nearly 150,000 annual visitors who have opportunities for hiking, boating, fishing, and camping. Visitors access the park via personal vehicles, boats, or non-motorized transport through a State Trail. Mixed pine uplands, jack pine (*Pinus banksiana*) barrens, and conifer bogs exist within the park. Black ash *(Fraxinus nigra)* is in the park’s lowland forests. EAB had not been detected in the park as of 2022.

### Questionnaire

A multi-section onsite questionnaire was developed, pre-tested, and implemented. Of interest were questions focused on visual preferences and displacement intentions in response to photos depicting select management approaches to an EAB infestation, EAB knowledge, and familiarity, perceived importance to manage EAB, as well as visit details.

**Fig. 1:**
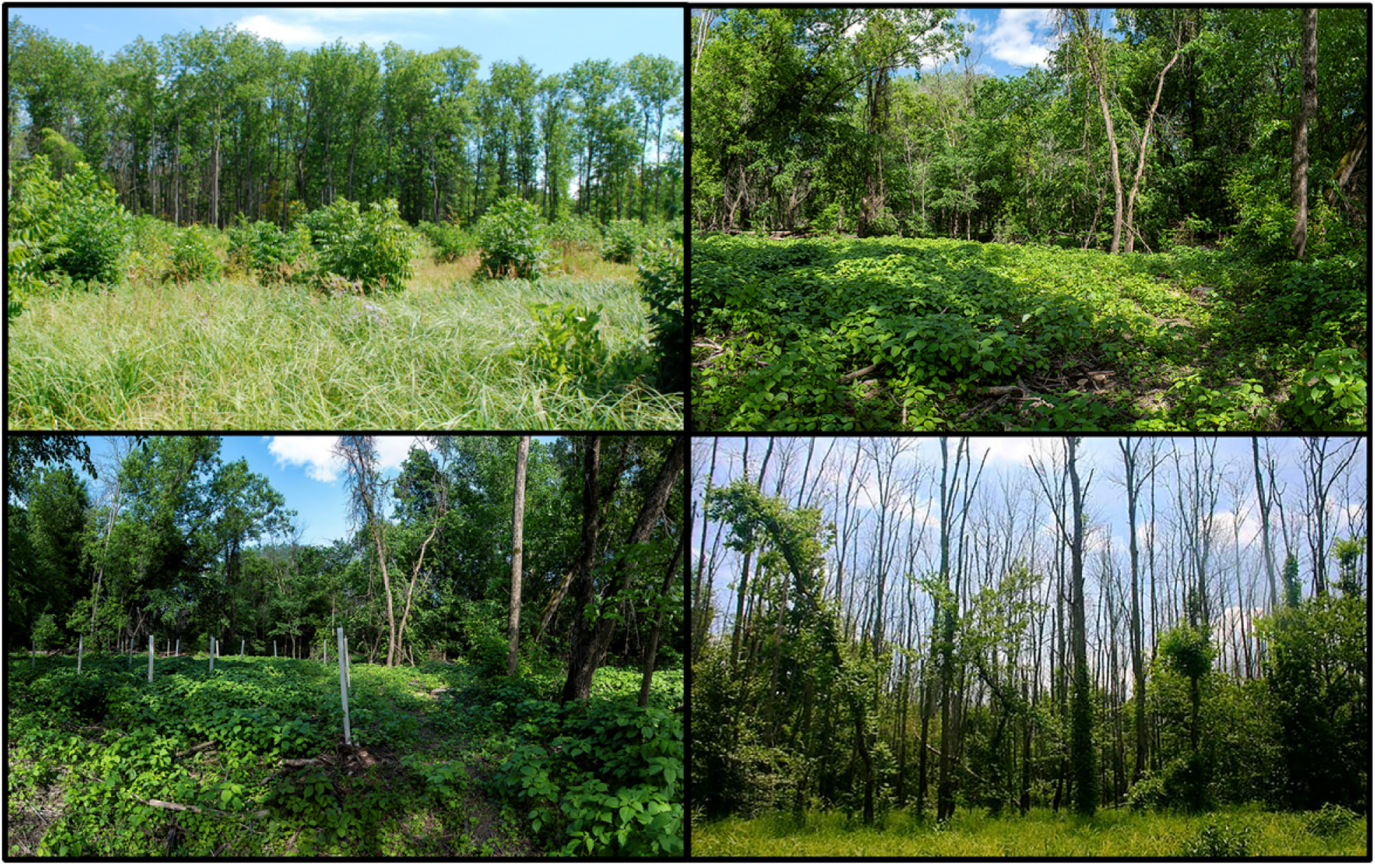
Landscape treatment images showing “complete harvest, natural regeneration” (top left); “select harvest, natural regeneration” (top right); “select harvest, planted trees” (bottom left); “do nothing” (bottom right)

Both preferences for and displacement intentions from an area were assessed in response to photos depicting landscapes resulting from the forest management approaches utilized for EAB: “do nothing,” “select harvest, natural regeneration,” “select harvest, planted trees,” and “complete harvest, natural regeneration” (Fig. 1). For preferences, visitors simply selected which of the landscapes they preferred. If respondents indicated they intended to displace or were unsure if they would displace from any of the landscapes, they were asked where they would go instead: an area within the site (intrasite displacement), an area outside the site (intersite displacement), stay home, or other. Photos were presented in random order and acquired through on-site visits of Minnesota forests about 5 years after management implementation.

A combination of variables assessed respondent EAB knowledge and familiarity. EAB knowledge was assessed on a 1–4 ordinal scale: 1 = never heard of it, 2 = heard of it but know nothing about it, 3 = heard of it and have some knowledge, and 4 = know a lot about it. Familiarity with the four selected management treatments was assessed on a 1–7 interval scale, with 1 = very unfamiliar and 7 = very familiar. Importance to manage forests in response to EAB was assessed on a 1–7 interval scale, with 1 = very unimportant and 7 = very important. Respondents’ personal experience with EAB was evaluated via three questions: if EAB resided on their property of residence, in their community, or if they have ever visited an area visually impacted by EAB. Experience options included a simple yes, no, or did not know. Visit information included first-time visitor status. Demographic information and experience with AR and VR were collected at the end of the questionnaire.

Respondents were randomly assigned to one of four informational interventions: 1) no management information (control), 2) text with photo display, 3) augmented reality (AR), and 4) virtual reality (VR). The message content was consistent across mediums but communicated through increasing levels of interactivity, engagement, and, subsequently, presumed central-route elaboration. In alignment with the ELM (Petty and Cacioppo 1986), all messages contained first-person words such as “we” and “you” to elicit a sense of personal relevance.

The photo/text intervention consisted of a four-page laminated informational sheet with photos and text call-out boxes about each management treatment (see supplemental materials). The AR intervention activated an interactive, 360° landscape image that shared the same information in the photo/text intervention, but the written words were spoken by a female voice with natural sounds in the background. Visitors were instructed to “click through” floating text boxes via a touchscreen that led to additional information accompanied by images or a video in each forest management approach (see supplemental materials). The VR intervention immersed the participant in the landscapes when they donned VR goggles attached to a supplied Android phone and, again, ‘clicked through’ informative text boxes to receive information about each treatment (see Supplementary materials). The VR intervention was even more immersive as it produced a more vivid, sensory-rich virtual environment by occupying the user’s full field of vision (Steuer 1992). The VR intervention was also more interactive as users could ‘follow’ a blue dot that served as their cursor to select informational text boxes, while users of the AR intervention selected text boxes via a touchscreen. Photos were taken through Nikon D850 and 810 with video acquired through 360 Cameras Insta360 Pro-8K and GoProMax. The VR app was developed and tested in Unity and the AR treatment in Zappar. The average completion time was 14 min.

### Data Collection

Sampling took place at commonly visited areas in each park. A stratified cluster sample of adult respondents (18 years or older) passing by researchers was systematically sampled. Within groups, the group member with the most recent past birthday was asked to participate.

A total of 746 visitors were invited to participate and more than half agreed (388 responses; 52% response rate). Non-respondents’ recreation activity, general EAB knowledge, first-time visitor status, and residential zip code were queried. Non-respondents knew significantly less about EAB (1.8%) than respondents (31.9%; Cramer’s *V* = 0.54; *X*^2^ (3, *N* = 698) = 199, *p* < 0.001) and were significantly more likely to engage in walking/hiking and dog walking.

### Data Analysis

Data were entered, cleaned, and analyzed via Statistical Package for Social Sciences (SPSS, version 26). A comparison of select key variables (age, recreation motivations, importance of managing forests in response to EAB, perceived problem that invasive species pose, and first-time visitor status) revealed only one site had differences: Wild River (first-time visitation and the perceived problem posed by invasive species). Therefore, site data were combined.

Chi-square tests assessed the relationship between message medium and preference or displacement intentions. Cramer’s *V* identified any significant effect sizes. As displacement was low (2.4–10.9% across sites) and those ‘unsure’ were considered impressionable via information, those who indicated “yes” and “unsure” about displacing were combined.

## Results

### Sample Characteristics, Motivations, and Experience with AR/VR

Respondents identified as primarily white (92%), non-Hispanic (97.4%) and were evenly distributed by age from 18 to 83 (avg: 47.5 years old). Seventy-two percent of respondents were repeat visitors and 43.5% traveled less than 10 miles from their primary residence to reach the study site(s). Fifty-eight percent were from the seven-county Minneapolis-St. Paul region and nearly 8% of respondents were from out of state. Respondents were generally inexperienced with AR and VR as nearly half had never used AR (49.2%) or VR (46.1%) and more than 40% reported using them less than ten times before the day we encountered them.

### EAB Knowledge, Prior Experience, Familiarity, and Importance to Manage Forests in Response to EAB

Over 75% of respondents indicated EAB awareness and possessed at least some knowledge of it; however, personal experience with EAB varied. Nearly equal percentages were aware they had visited an area infested with EAB as were unsure (39.1 and 37.8%). Nearly one-quarter of respondents were unaware if EAB existed on their property (23.5%) and half of the respondents (50.8%) indicated they had EAB in their community while 34.2% were unsure.

Most visitors believed that managing forests in response to EAB was important or very important (64%), however, visitors were generally unfamiliar with management strategies. Less than 25% were familiar or very familiar with any of the strategies listed: doing nothing (22.7%), select harvests (13.1–16.3%), and complete harvest (10.6%).

### Landscape Preferences

**Fig. 2:**
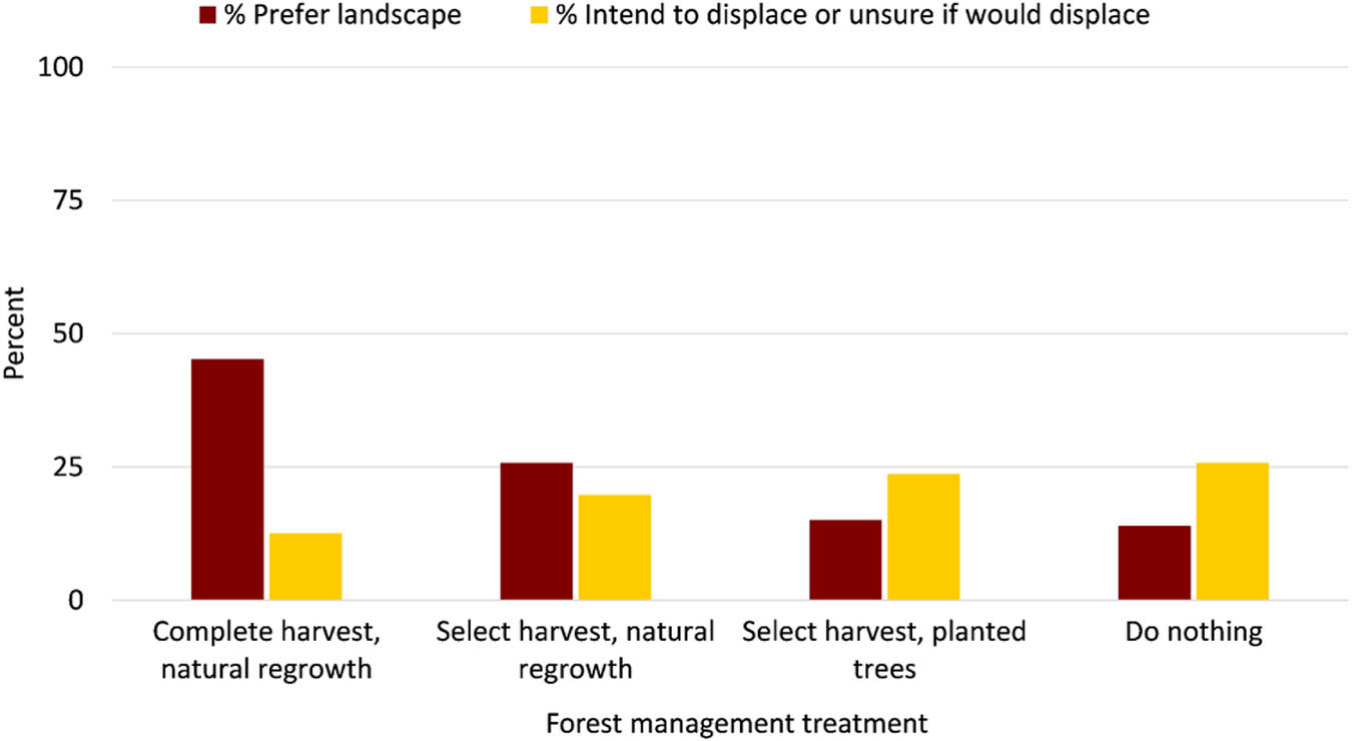
Percentage of respondents who indicated which landscape photo they visually preferred and whether they intend to displace from the landscapes pictured (received no information, *n* = 93–97)

Without any information, visitors most preferred photos depicting complete harvest with natural regeneration (45.2%) and least preferred photos depicting doing nothing (14%; Fig. 2). Message medium was significantly related to landscape preferences in three of the four landscapes X^2^ (df 9, *N* = 371) = 30.9, *p* < 0.001, Cramer’s *V* = 0.17; Table 1). For complete harvest scenarios, those receiving AR and VR information expressed significantly greater landscape preference for them compared to respondents viewing only photos, but not differently from those who received no information (AR and VR: 51.1%; Photo/text: 31.5%; No information: 45.2%). For select harvest treatments with planted trees, respondents who read and viewed the photo/text expressed increased landscape preference over no information (32.6 vs 15.1%) whereas for select harvest with natural regeneration, those who engaged with VR had significantly lower preferences than any of the others, including no information (16–25 vs 4.3%). Preferences remained low for the “do nothing” landscape regardless of any information provided.

**Table 1:** Percent of respondents preferred landscape encountering forested landscapes managed in response to emerald ash borer (*n* = 371)

	Preference %			
Forest management treatment	Definition only (*n* = 93)	Photo/text (*n* = 92)	Augmented (*n* = 94)	Virtual (*n* = 92)
Complete harvest, natural regrowth	45.2^a, b^	31.5^b^	51.1^a^	51.1^a^
Do nothing	14^a^	10.9^a^	9.6^a^	19.6^a^
Select harvest, planted trees	15.1^a^	32.6^b^	23.4^a, b^	25^a, b^
Select harvest, natural regrowth	25.8^a^	25^a^	16^a^	4.3^b^
Total	100	100	100	100

Values with different superscript letters within a row are significantly different (*p* < 0.05)

### Displacement Intentions

Without any information, intentions to displace from landscape photos ranged from 12.6 to 25.8% (Fig. 2). Respondents were most likely to displace in response to photos representing “select harvest, natural regeneration” (25.8%) and least likely to displace from photos representing “complete harvest, natural regeneration” (12.6%). Nearly 20% indicated they would displace or were unsure if they would in response to “do nothing,” followed by “select harvest, planted trees” (23.7%). Among those displaced, intrasite displacement was the most common (52–66.6%) followed by intersite displacement (32–43.5%). Very few, if any, respondents indicated they would stay home in response to the four landscapes (0–4%).

Message medium was significantly and moderately related to displacement intentions in one of four photos: “select harvest, planted trees,” X^2^ (df 3, *N* = 382) = 16.6, *p* = 0.001, Cramer’s *V* 0.21; Table 2) where any information significantly reduced displacement intentions.

**Table 2:** Percent of respondents displacing or unsure of displacement in response to forested landscapes managed in response to emerald ash borer (*n* = 382–384)

	Displace/unsure combined %			
Forest management treatment	Definition only (*n* = 95–97)	Photo/text (*n* = 92–100)	Augmented (*n* = 95)	Virtual (*n* = 92)
Complete harvest, natural regrowth	12.6^a^	9^a, b^	6.3^a, b^	4.3^b^
Do nothing	19.8^a^	26^a^	26.3^a^	20.7^a^
Select harvest, planted trees*	23.7^a^	8.2^b^	6.3^b^	10.9^b^
Select harvest, natural regrowth	25.8^a^	24^a^	35.8^a^	30.4^a^
Total	100	100	100	100

Values with different superscript letters indicate difference between columns, treatments with * indicate significant association (*p* < 0.05)

## Discussion

Onsite visitor questionnaires in a U.S. Midwest state assessed if and how increasingly engaging message mediums related to state park visitor landscape preferences and displacement intentions: specifically, messages that communicated about management in response to EAB. While messaging impacted preferences for three of the four treatments, significant changes in displacement intentions emerged in only one of the four. Implications, study limitations, and opportunities for future research follow.

The preferred landscapes mirror past research where open forest structures, the presence of ground vegetation, and homogenous ground texture are preferred (Ribe 1990; Daniel and Boster 1976; Kaplan and Kaplan 1989; Kaplan et al. 1989; Ribe 1990; Ulrich 1983) and presence of tree death, downed wood and single species are not (Ribe 1990; Ryan 2005; Schneider et al. 2019).

Displacement intentions in this study were rather low compared to the other limited research related to terrestrial pests (~30–68%; Schneider et al. 2019). In line with landscape preference research, visitors were most likely to displace from landscapes containing commonly disliked elements: bare soil, standing and ground-laden dead wood, and large canopy gaps (Edwards et al. 2012; Ribe 1990). However, whereas past research revealed landscapes depicting regeneration harvests were strongly disfavored and incited the most displacement (Kearney, 2001; Schlueter and Schneider 2016; Schneider et al. 2019), visitors were least likely to displace from the complete harvest natural regeneration harvest landscape presented in this study. One explanation is that photos in this study depicted landscapes five years after their treatment, allowing the landscape to regenerate and mitigating the undesirable elements. Indeed, past research indicated scenic beauty increases with time since harvest (Brunson and Reiter 1996; Hull and Buhyoff 1986). In contrast, other photo-based studies assessing responses showed respondents immediate outcomes of the complete harvest scenarios with no regeneration (Kearney 2001; Schneider et al. 2019).

Related to information impacts, findings partially support previous research that messages incorporating the ELM, when used in an outdoor recreation context, can promote issue-relevant thinking and influence preferences and behavior intentions (Lazard and Atkinson 2015; Miller et al. 2019; Vezeau 2014). Possible reasons for the lack of consistent significant influence relate to external, recipient, and source factors that may have impeded respondents’ ability and/or motivation to elaborate on the presented message arguments. External factors may have interfered with the visitors’ ability to elaborate at the level encouraged by the interventions. Distractions impact one’s ability to elaborate on a message and result in less favorable attitudes toward strong messages; additionally, high levels of distractions negatively impact one’s ability to recall message arguments (Petty and Cacioppo 1986; Slater and Steed 2000). External factors here include fellow group members attempting to interact with the respondent, the occasional presence of biting insects, background noises, and surrounding sights. A variety of recipient factors play into one’s ability to elaborate on message arguments including knowledge, relevance, and motivations. The general lack of familiarity surrounding the management treatments may have impeded respondents’ ability to draw on prior knowledge and elaborate on the message arguments (Haugtvedt and Wegener 1994). The lack of personal EAB experience and familiarity with management treatments may indicate that EAB management, though important, was not personally relevant to some participants, impacting their elaborative motivation (Celsi and Olson 1988).

Message source factors are another important factor, particularly message comprehensibility and source-initiated distractions (Petty and Cacioppo 1986). In the context of technological interventions, ease of use with interactive materials is important. If an intervention is too interactive or difficult to use, users become frustrated and associate such feelings with the message (Liu and Shrum 2009). While the interventions in this study were pre-tested to optimize use, nearly half of the respondents had no prior AR or VR experience, and some first-time users may have had trouble attaining a sense of presence (Sagnier et al. 2020).

Management implications include the importance of using information to communicate forest management and the need for critical evaluation of advanced communication technologies. With no information, nearly one-quarter of visitors may displace from selective regeneration harvests with planted tree landscapes and not prefer them; however, providing the public with even just photo and text information about the treatment can significantly decrease the displacement intention but not landscape preference.

Information impacted preferences and selectively reduced displacement intentions by up to 15%. As the AR and VR interventions did not significantly decrease displacement intentions compared to the photo/text intervention, the investment in more dynamic technological communication materials may not be needed to influence visitor preferences and displacement in all settings. In contrast to past studies, the multisensory elements did not significantly impact revisit intentions (Guo et al. 2021; Alyahya and McLean 2021). More broadly and as identified in previous research, minimizing undesirable visual features in forest recreation viewscapes (Edwards et al. 2012; Ribe 1990) remains important.

### Limitations and Future Research

Limitations exist in this study. First, sampling was conducted at three state parks all located in one midwestern U.S. state in the summer. And, while the sample reflected the state park visitor population, it was less diverse than the state’s population. As such, efforts to understand visitors with multiple racial and ethnic backgrounds seem imperative. Second, visitor behavioral responses are not limited to summer recreationists and other seasons should be considered, as Kaae (2000) found that timber harvests impacted the visual quality of a winter recreation site and negatively influenced displacement intentions. Third, displacement intentions and actual displacement behaviors were compared. While behavioral intentions are a useful component in understanding behaviors (Ajzen 1991), observing actual behaviors at the moment is the most reliable method to inform managers about displacement; however, documenting this behavior can be difficult and time-intensive (Schneider and Budruk 1999).

Future work could expand on the immersion elements and conceptual connections. One such case includes enhancing or incorporating additional audio and visual cues, such as exploring different narrative styles. For example, Kang and Gretzel (2012) found that multiple voices speaking in a conversational style within a message increased recipients’ social presence. Implementing an informational game may be of interest as they can positively influence knowledge and stewardship behaviors pertaining to invasive species (Howard 2017). To confirm if the AR and VR interventions promoted heightened levels of central-route elaboration, post-intervention elaborations are suggested (Miller et al. 2019; Shen and Seung 2018). Conceptually, considering other responses such as acceptance would be of interest, given the differences illustrated to date (Arnberger et al 2017; Manning and Freimund 2004; Schneider et al. 2019) and place attachment, which is impacted by perceived visual quality (Kaae 2000).

Clearly, information positively influences visitor preferences and intended behaviors, but the extent of engagement required varies. Further research can elaborate on these findings and continue to refine the relative merit of augmented and virtual reality in informational efforts related to natural resource and invasive species management.

Insert Appendix A Example of the photo/text intervention for the landscape treatment “select harvest, planted trees.”

Insert Appendix B “Zappar codes” used to access landscape treatments via augmented reality. Codes are scanned via an Android smartphone while using the “Zappar” app and reveal one of the four landscape treatments, depending on which code is scanned. Created using Microsoft Word.

## Supplementary information

Supplementary Material 1

Supplementary Material 2
