# Supplementary figures and images for: What Really Works? Testing Augmented and Virtual Reality Messaging in Terrestrial Invasive Species Management Communications to Impact Visitor Preferences and Deter Visitor Displacement
Source: Environ Manage. 2023 Jan 16;71(6):1199–212. doi: 10.1007/s00267-023-01787-z (PMC10183427; doi:10.1007/s00267-023-01787-z)

**
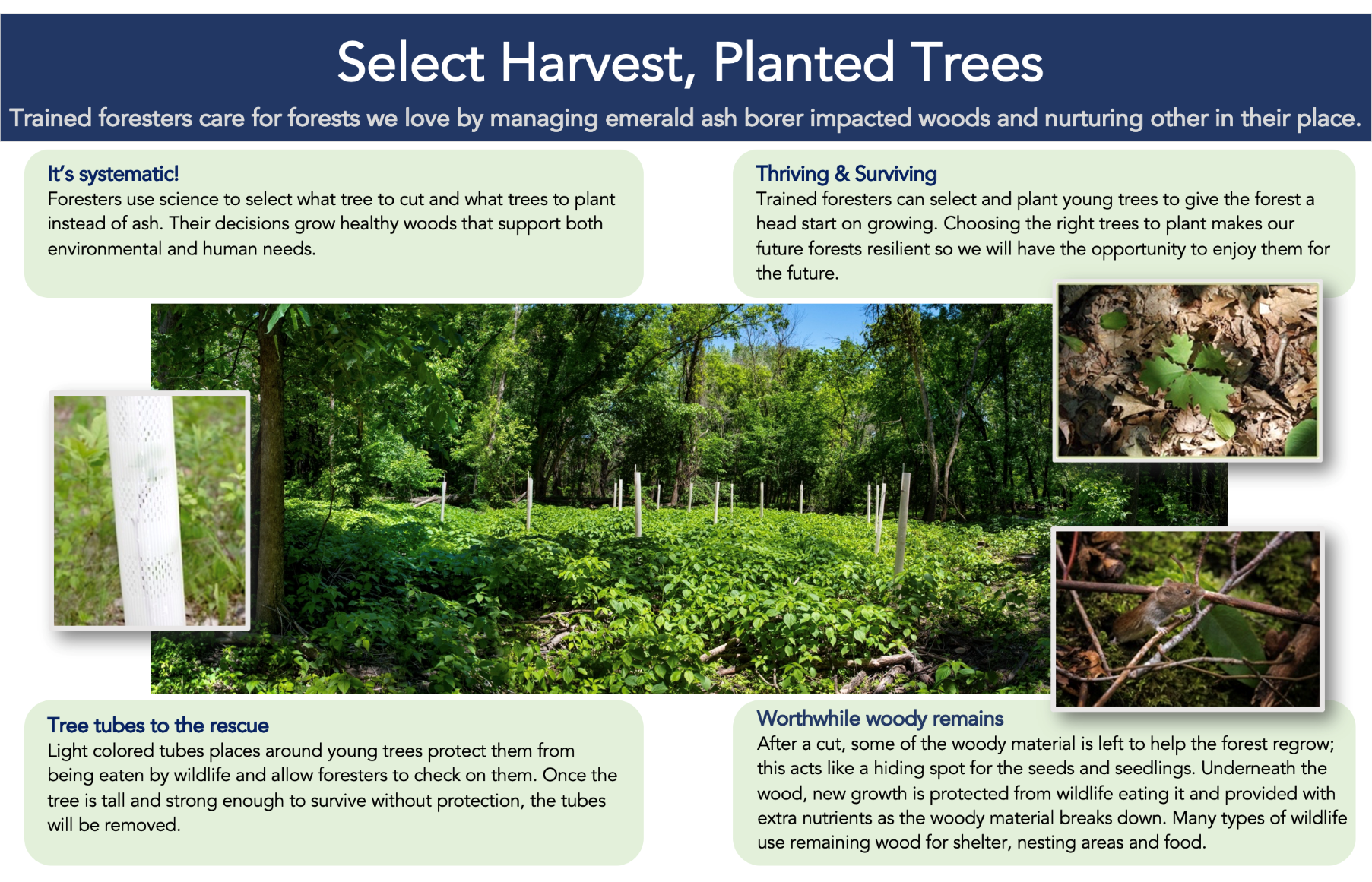
**

Supplement: Supplementary file 1 — Supplementary Material 1 [file 267_2023_1787_MOESM1_ESM.docx]

**
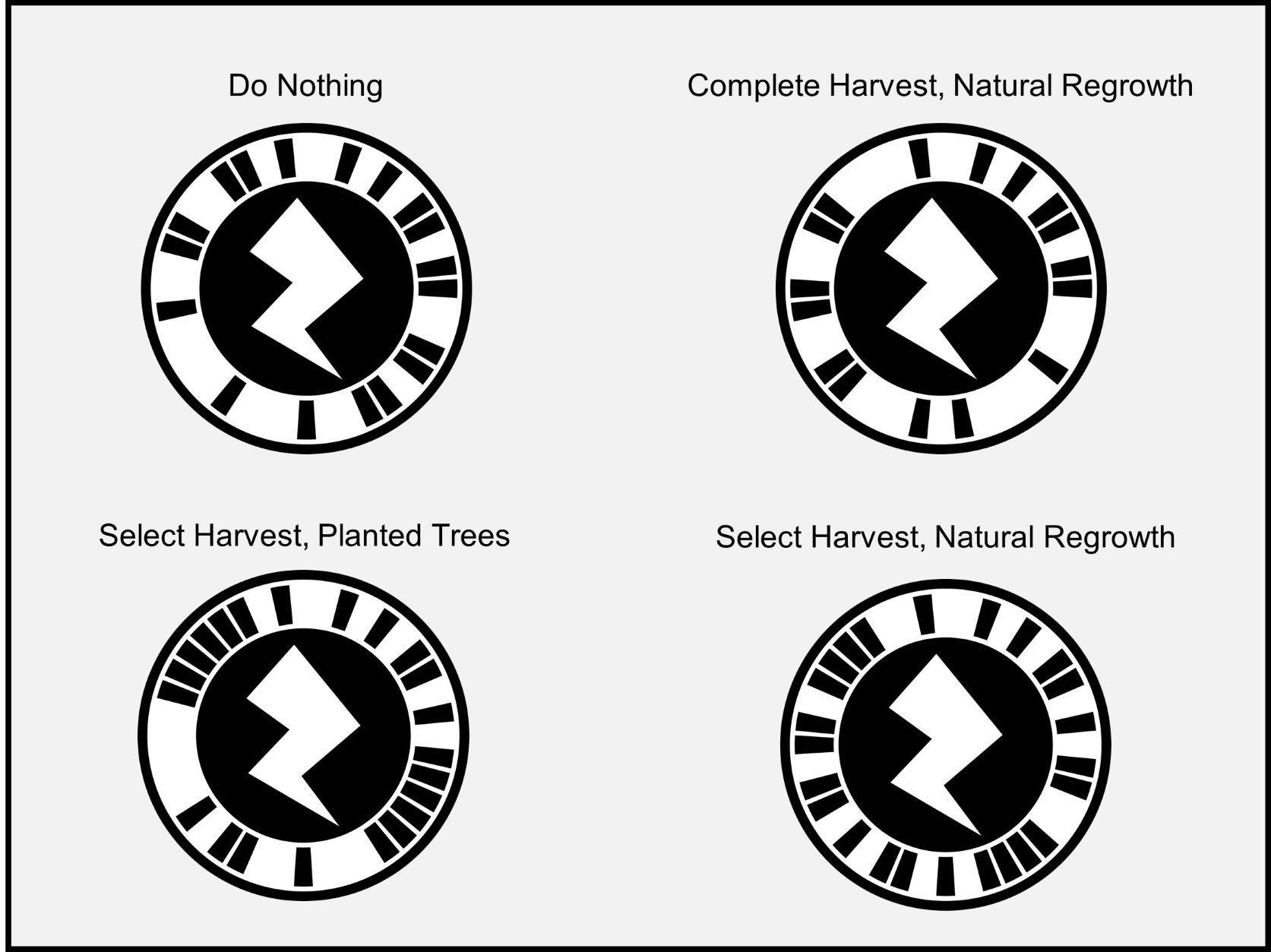
**

Supplement: Supplementary file 2 — Supplementary Material 2 [file 267_2023_1787_MOESM2_ESM.docx]
